# Small Pilot Survey on Parents’ Perception of the Relationship between Children and Pets

**DOI:** 10.3390/vetsci4040052

**Published:** 2017-10-16

**Authors:** Natalia Russo, Diana Vergnano, Domenico Bergero, Liviana Prola

**Affiliations:** Department of Veterinary Science, University of Turin, 10095 Grugliasco, Italy; natalia.russo@unito.it (N.R.); domenico.bergero@unito.it (D.B.); liviana.prola@unito.it (L.P.)

**Keywords:** children, parent perception, pets

## Abstract

Since companion animals are taking on more important roles in family life, the aim of this study was to evaluate the perception of parents about the relationship between their children and pets. A number of parents were asked to fill in a questionnaire; the principal topics were: pet ownership, pet care, relationship between pets and children, and sources of information about pet management. Eighty-two parents completed the survey; 71.4% of them already had pets before having children; pet care and health has emerged to be rather important, since 96.4% of the pets are taken to the veterinarian at least once a year; moreover, the great majority of the parents (97.2%) were not worried about the possible risks, linked to pets, pertaining to their child’s health. The present survey confirms that pets are mostly considered as members of the family, and not only as a benefit for the children. Moreover, the relationship between children and pets is basically seen as a positive experience for children.

## 1. Introduction

The number of pets in Italy has been estimated to be around 60 million [1], while there are about 8.1 million children under 14 [2]. It has emerged, from recent literature, that the role of pets has changed over the years. According to Walsh (2009) [3], companion animals are an important part of family life and are often treated as family members. Furthermore, different studies have pointed out the role that pets play in people’s health [4,5,6]. However, most of these studies have focused on adults, while much less is known about the role of companion animals related to children in the family environment. On one hand, pets are thought to give both physiological and psychological health support, but on the other hand, they can represent a risk as far as bites and zoonosis are concerned [7,8]. A few studies have reported the perceptions of the children themselves about having a pet [9,10]. To the best of the authors’ knowledge, there is only one study about the parental perception of the relationship between pets and their children [11] and it focused on play and care behaviors. Raising a child with pets can lead to many benefits; however, having a pet in the family is not always easy, since many problems can emerge, and families do not always have the right knowledge to manage them. Therefore, the aim of our work was to investigate how pets are perceived in Italian families and especially how the relationship between the children and their pets is perceived by the parents.

## 2. Materials and Methods

### 2.1. Data Collection and Selection of the Participants

A questionnaire was drawn up. The questionnaire was distributed online using social networks. It was addressed to parents of children under 14 years of age. Moreover, the parents had to own one or more pets, or at least have planned to have a pet in the future. Before sending in the questionnaire, the participants had to answer all the questions.

### 2.2. Questionnaire Setup

The survey was composed of 16 multiple-choice questions, which are reported in Table 1. The questions covered the following themes: pet ownership & animal care, parent-pet relationship, child-pet relationship, and health risks linked to pets. Furthermore, the parents were asked whether they believed that they had all the information necessary to correctly manage the relationship between their pet and their children and whether they would ask or had asked for the veterinarian’s advice before choosing their pet.

### 2.3. Statistical Analysis

A descriptive statistic has been proposed for this pilot study. The answers were tabulated using a commercial program (Microsoft Excel 2010, Microsoft, Redmond, WA, USA) and the results were expressed as percentages.

## 3. Results

A total of 110 questionnaires were sent out, of which 82 parents completed the survey.

A total of 86.1% of the parents had pets: 52% of them only owned a dog, 16% only owned a cat, and 32% owned several animals. As far as the questions about animal care is concerned, 56.1% of the parents declared they fed their pets commercial diets (3.7% of them searched for the most natural commercial feeds); 36.6% instead fed them a mixed diet, while 7.3% fed homemade food. A total of 84.1% of the families treated their pets for external parasites. Most pets (62.2%) were taken to the veterinarian once a year, 25.6% were taken twice a year, 8.6% three or more times per year, and 3.6% were never taken. A total of 51.2% declared they asked a veterinarian for advice about their choice of animal.

A total of 72% already had a pet before having children. Furthermore, the majority of the participants (71.9%) declared that their relationship with their pet had not changed after the arrival of the baby; in 56.5% of the cases in which the relationship had changed, it had improved. The relationship between the children and the pets was considered positive by all of the parents (70.7% stated the pet was a playmate, while 29.3% declared it was someone to take care of). The great majority of the parents (97.5%) declared they were not worried about the possible health risks for the children linked to the presence of animals: in fact, only 1.2% of the parents were worried about hygiene and 1.2% about sanitary risks. Moreover, most had not increased the number of visits to the vet after their child’s birth (only 3.7% had). Instead, 80.5% of the children shared the same rooms with the animals, even though only 30.5% slept with them.

Finally, 90.2% of the parents declared they did not need any further information about the child–pet relationship.

## 4. Discussion

In our study, the percentage of parents with a household pet, 86.1%, is higher compared to percentage found by other authors [12] where parents with a pet where 54.8% (*n* = 746); anyway, the difference could also be due to the different sample size In our data, the percentage of families that owned only a dog (52%) was higher than the percentage that owned only a cat (16%) and the percentage that owned several animals (32%). In another study developed in Italy in 2008 by Slater and colleagues [13], the percentage found for cats was similar, while that found for dogs was lower: 15% had cats (60/397) and 33% (130/397) had dogs. The higher percentage of dogs in families with children could be due to the behavioral characteristics of this species, which makes them more suitable for interacting with children, especially small children.

The majority of the families (59.8%) only fed their pet commercial diets. This finding is in agreement with the Assalco survey 2017 [1], which reports that 77% of the interviewed use commercial feeds. Pet’s health seems to be quite important: 96.4% of the pets were taken to veterinarian at least once a year, and 84.1% of the families treated their pets for parasites. 

Regarding the child–pet relationship, the present survey shows that pets are generally considered as family members and not only as a benefit for the child, since, in the majority of the cases (71.4%), the pet was acquired before the birth of the child. The animals are not considered dangerous for the child’s health and they are perceived to have a role in improving relationship capacities and responsibility. This is in agreement with what Catcher and Friedman stated in 1982 [14]. They suggested seven main roles for pets, including companionship and someone to care for.

A total of 90.9% of the parents that were interviewed believed they already had all the information necessary about the relationship between children and pets; nearly half of the parents asked their veterinarian for advice about the choice of animal, but no information is available about the other sources. It would be interesting to focus more on this point in order to verify whether these sources are reliable and complete.

## 5. Conclusions

In conclusion, the antenatal and postnatal presence of furry household pets has been demonstrated to confer protection against development of atopy, food sensitization, and metabolic disease [15,16,17] in children and has been linked to reduction of cardiovascular risk in infants [18]. With this study, we gave stimulating points for reflection, even if the sample was small, because the presence of household pets highlighted by our data is high compared to other data and could be interesting to investigate with a multidisciplinary approach, if it means lower presence of the above mentioned diseases in children population in the same area. It would be useful, therefore, to extend this survey to a bigger population in different parts of Italy and to investigate if there are differences between pet species (cat vs. dog).

## Figures and Tables

**Table 1:** Survey.

**1. Do you have any pets?** □ Yes □ No	**9. If so, how did the relationship change?** □ It is better now, my pet receives more attention □ It is worse now, the time spent together has decreased □ It is worse now, I am worried when my child plays with our pet
**2. What kind of animal do you have/would you like?** □ Dog □ Cat □ Dogs and cats	**10. How do you consider the relationship between your child and your pet?** □ Positively, they are playmates □ Positively, the child learns/is learning to take care of someone □ Negatively, it can be risky
**3. How do you feed your pet(s)?** □ Commercial diet □ Homemade diet □ Mixed diet (commercial + homemade diets) □ Commercial diet, but as natural as possible	**11. Are you worried about the health of your child as a result of owning a pet?** □ Yes □ No
**4. Do you treat your pet for external parasites?** □ Yes □ No	**12. If so, what are you most worried about?** □ Bites and scratches □ Hygiene issues □ Health risks (parasites, infections…)
**5. How many times per year do you take your pet to the veterinarian?** □ Once □ Twice □ Three times or more □ Never	**13. Since the birth of your child, have you increased the number of veterinary appointments?** □ Yes □ No
**6. Would you ask for your veterinarian’s advice before choosing your pet?** □ Yes □ No	**14. Do your children and pets share the same spaces?** □ Yes □ No
**7. When your child was born, did you already have a pet?** □ Yes □ No	**15. Do they sleep together?** □ Yes □ No
**8. If so, when your child was born, did your relationship with your pet change?** □ Yes □ No	**16. Do you think the information you have about the management of your pet with regard to the relationship with your child is sufficient?** □ Yes □ No
